# Human acellular amniotic membrane loaded with hair follicle-derived cells promotes wound repair and appendage-like structure formation

**DOI:** 10.3389/fcell.2026.1947836

**Published:** 2026-10-01

**Authors:** Yanxin Jiang, Zhongwen Hou, Xichao Jian, Zeyao Tang, Yan Zhou, Xing Hu, Chengliang Deng, Shune Xiao

**Affiliations:** Department of Burns and Plastic Surgery, Affiliated Hospital of Zunyi Medical University, Zunyi, China

**Keywords:** biological scaffold, dermal papilla cells, hair follicle stem cells, human acellular amniotic membrane, wound healing

## Abstract

**Background:**

Deep skin injuries often heal with scarring and lack skin appendage regeneration, posing a significant clinical challenge. This study aimed to develop an hAAM-based combinatorial strategy for co-delivery of human hair follicle stem cells (HFSCs) and dermal papilla cells (DPCs) to full-thickness skin wounds, and to evaluate its effects on wound repair and appendage-like structure formation.

**Methods:**

hAAM was prepared and characterized. HFSCs and DPCs were isolated from human hair follicles and identified. The composite construct (hAAM + HFSCs + DPCs) was applied to full-thickness skin wounds in nude mice. Wound healing rates were monitored. Histological and immunohistochemical analyses were performed to assess re-epithelialization, collagen deposition, the presence of CD31-positive vascular structures, cell proliferation, fibrosis-related markers, and morphological features suggestive of appendage-like structures.

**Results:**

The hAAM scaffold co-loaded with HFSCs and DPCs was associated with accelerated wound closure, tissue repair, and altered expression of fibrosis-associated markers, including TGF-β and α-SMA, together with altered collagen deposition. The treatment was also associated with the appearance of PPAR-γ-positive structures with morphological features resembling sebaceous glands, as well as epithelial bud-like structures morphologically suggestive of early follicular development, in a subset of specimens. Although these morphological observations are preliminary and require further characterization with additional lineage-specific markers, they represent preliminary morphological observations of appendage-like structures.

**Conclusion:**

The hAAM scaffold co-loaded with HFSCs and DPCs was associated with accelerated wound closure, tissue repair, and altered extracellular matrix remodeling in a murine full-thickness wound model. Structures with morphological features resembling sebaceous glands and epithelial bud-like structures suggestive of early follicular development were observed in some samples; however, their cellular identity, maturity, and functionality remain to be determined. Further lineage-specific characterization and long-term functional studies are required to establish whether this strategy can promote appendage-like structure formation and functional skin restoration.

## 1 Introduction

The skin is often damaged by acute and chronic wounds, such as trauma, burns, diabetic ulcers, and venous stasis ulcers, which not only cause physical and psychological distress to patients but also imposes a substantial socio-economic burden (Nussbaum et al., 2018; Hu et al., 2023). Wound healing is a dynamic, orderly, and intricate process that plays a vital role in maintaining normal skin tissue function. It is typically divided into four interconnected and overlapping phases: hemostasis, inflammation, proliferation, and remodeling (Tu et al., 2021; Zhou et al., 2021; Peña and Martin, 2024). In contrast, abnormal wound healing, such as hypertrophic scarring or chronic ulceration, can lead to significant morbidity (Zheng et al., 2018). Currently, some dressings are available for wound repair, yet they exhibit limitations such as restricted therapeutic efficacy and the potential for secondary injury due to frequent dressing changes. Additionally, surgical interventions are applicable for wound repair in certain patients; however, these procedures are often associated with significant patient discomfort, high costs, and the risk of postoperative scar formation, which can adversely affect the patient’s quality of life (Qi et al., 2022). Consequently, wound management has garnered increasing attention in recent years, with effective promotion of wound healing emerging as a key research priority. In recent years, the development of implantable bioengineered skin has diversified, encompassing epidermal, dermal, and composite types, among which some products have now been approved for human clinical use (Roy et al., 2024). Furthermore, substantial progress has been made in various experimental approaches integrating stem cell therapy into skin wound healing (Li et al., 2019; Farabi et al., 2024).

The hair follicle, as a cutaneous appendage, is easily accessible and contains various stem cells such as hair follicle stem cells and mesenchymal stem cells. Hair follicle-derived stem cells can continuously self-renew, differentiate, regulate hair growth, maintain skin homeostasis, and demonstrate considerable advantages in wound healing, holding promising clinical application prospects (Li et al., 2019; Heidari et al., 2023; Wang et al., 2020). Upon activation, HFSCs rapidly proliferate, migrate to the wound site, and differentiate into epithelial cells (Joost et al., 2018). HFSCs possess a long lifespan and high plasticity (Yamane et al., 2021). Compared to other types of adult stem cells, HFSCs offer the advantages of easy accessibility, immunomodulatory properties, non-tumorigenicity, and absence of ethical concerns, making them ideal candidate stem cells for regenerative medicine (Obara et al., 2025). Studies have found that HFSCs accelerate wound healing by promoting rapid re-epithelialization and vascularization (Quan et al., 2017; Mao et al., 2022). Furthermore, HFSCs play a significant role in maintaining epithelial homeostasis and turnover (Babakhani et al., 2020). Beyond differentiating into skin epidermal cells, HFSCs can also multipotently differentiate into endothelial cells, hair follicle epithelial cells, sebaceous gland cells, and various other cell types with specialized functions *in vivo* (Li H. et al., 2022). However, when used alone, hair follicle stem cells lack specific inductive signals. Upon transplantation into the inflammatory and rapid re-epithelialization-dominated wound microenvironment, these cells tend to differentiate into keratinocytes for epidermal repair or form cyst-like structures, rather than achieving autonomous and orderly assembly into complex hair follicle-sebaceous gland units. Alam et al. (Alam et al., 2019) also reported that hair follicle stem cells transplanted into wounds tend to participate in the wound repair process rather than initiate *de novo* hair follicle formation. Hence, how to provide an appropriate microenvironment and critical inductive signals has become a central challenge for promoting appendage-like structure formation.

DPCs located at the base of the hair follicle, are specialized mesenchymal cells that can differentiate into multiple cell lineages under specific conditions (Wang et al., 2020). Previous studies have shown that DPCs regulate HFSC activation, vascularization, and hair follicle morphogenesis through epithelial–mesenchymal interactions and paracrine signaling (Chen et al., 2020; Li J. et al., 2023). Research has shown that DPCs effectively promote wound healing by facilitating the repair of dermal components and secreting various growth factors and cytokines (Madaan et al., 2018; Gharzi et al., 2003; Leirós et al., 2014). Crucially, both HFSCs and DPCs serve as core seed cells for hair follicle reconstruction (Madaan et al., 2018; Joulai et al., 2017). Hair follicle morphogenesis and the hair cycle result from the interaction between HFSCs and the underlying DPCs (Joulai et al., 2017). DPCs possess hair follicle-inductive capacity, enabling them to induce hair follicle morphogenesis and regulate hair follicle development and cycling (Madaan et al., 2018). Previous studies have demonstrated extracellular vesicles (EVs) from DP cells promoted faster epithelialisation and increased vascularization in mice with full-thickness wounds (Wang et al., 2023). Moreover, DPC exosomes (DPC-Exos) can expedite wound healing and stimulate hair follicle regeneration (Shang et al., 2024). However, dermal papilla cells tend to lose their inherent aggregative behavior and inductive capacity in monolayer cultures *in vitro*, and it remains difficult to maintain their functional phenotype long-term after transplantation. As a result, their standalone application often fails to achieve stable regeneration of tissue structures.

Thus, the combined use of hair follicle stem cells and dermal papilla cells is expected to exert combined effects and compensate for their respective limitations. HFSCs provide epithelial progenitors, whereas DPCs provide inductive mesenchymal signals. Their combination may recreate epithelial–mesenchymal interactions and improve wound repair outcomes (Hasegawa-Haruki et al., 2024). The co-transplantation of these 2 cell types can mimic the core microenvironment of epithelial-mesenchymal interactions within the hair follicle unit, thereby systematically creating a microenvironment conducive to appendage-associated regeneration. This strategy holds particular promise for promoting the formation of appendage-like structures. The reconstruction of these structures is not only crucial for restoring skin barrier function but also represents a core aspect of improving wound healing quality—an ongoing challenge in clinical treatment to this day.

However, cell transplantation is confronted with several challenges, including low retention rates, poor survival, and difficulties in achieving effective engraftment and functionality at the wound site. Therefore, selecting an appropriate biological scaffold as a carrier is crucial for cell delivery, protection, and functional performance. Decellularized biomaterial-based wound dressings, as natural materials, are increasingly favored in the field of regenerative medicine and have garnered extensive research interest (Wang et al., 2021; Li Y. et al., 2023). Among these, human amniotic membrane stands out due to its easy availability and freedom from ethical concerns. hAAM as a natural biological scaffold, exhibits low immunogenicity along with demonstrated anti-infective and anti-scarring properties (Henry et al., 2020). It can serve not only as a clinical dressing for skin wounds but also as a tissue engineering scaffold for delivering drugs or functional cells (Xiao et al., 2021). Our previous studies have utilized hAAM to load adipose-derived mesenchymal stem cells derived exosomes (ADSC-Exos), and this composite scaffold has shown significant potential in promoting diabetic wound healing (Xiao et al., 2021). Therefore, we hypothesize that hAAM may serve as an effective carrier for delivering hair follicle-derived cells to skin wounds, thereby enhancing the bioavailability of these cells, accelerating the wound healing process, improving the quality of healing, and improving the quality of healing.

Although conventional skin cells such as keratinocytes and dermal fibroblasts are more readily available and expandable, our rationale for selecting HFSCs and DPCs was to investigate whether a combination of epithelial progenitor cells and hair-inductive mesenchymal cells could provide a microenvironment more relevant to cutaneous appendage-associated regeneration. However, whether conventional keratinocyte/fibroblast combinations can achieve comparable wound-healing or appendage-associated outcomes remains an important question that was not directly addressed in the present study. Therefore, this study explores whether an hAAM-based combinatorial strategy for co-delivery of HFSCs and DPCs can overcome the limitations of single-cell applications and promote wound healing outcomes, including altered fibrosis-associated marker expression and early observations of PPAR-γ-positive gland like structures and epithelial bud like structures morphologically suggestive of early follicular development. Although HFSC/DPC-based approaches and hAAM-based cell delivery have been investigated separately, the specific novelty of this study lies in combining these two components to recreate epithelial–mesenchymal interactions within the wound microenvironment. We acknowledge that functional regeneration has not been demonstrated in the present study, and our findings are presented as preliminary observations warranting further investigation.

## 2 Materials and methods

### 2.1 Animals and ethical approval

A total of 50 male BALB/c nude mice (6–8 weeks old) were obtained from Gem Pharmatech (Jiangsu) Co., Ltd. They were observed in the quarantine room of the SPF Animal Experimental Center of Zunyi Medical University (SYXK (Qian) 2021–0004) for 1 week without abnormalities before being transferred to the animal housing facility. Human tissue specimens, including human amniotic membranes (hAMs) and human scalp hair follicles, were collected with written informed consent obtained from each participant. Human amniotic membranes (hAMs) were collected from three healthy women undergoing cesarean sections at the Affiliated Hospital of Zunyi Medical University. Normal human scalp hair follicles were obtained from three independent donors during hair transplantation procedures. All experimental protocols were approved by the Ethics Committee of the Affiliated Hospital of Zunyi Medical University (Application No. ZMU (2022)1-149). Additionally, all animal experiments were approved by the Institutional Animal Care and Use Committee of Zunyi Medical University (Application No. ZMU21-2202-094) and conducted in accordance with the guidelines set forth by the National Health and Medical Research Council of China. This study was conducted in strict accordance with the ARRIVE 2.0 guidelines. All efforts were made to minimize animal suffering and reduce the number of animals used. The method of euthanasia at the end of the experiment was cervical dislocation.

### 2.2 Isolation and identification of human HFSCs and DPCS

Primary human HFSCs were purchased from Wanwu Biotechnology Co., Ltd. (Hefei, China) (The donor selection, tissue procurement, and cell isolation procedures were performed by the supplier prior to purchase and were not part of the present study) and cultured in DELF primary keratinocyte culture medium (Wanwu Biotechnology Co., Ltd., Hefei, China), which consists of primary keratinocyte basal medium supplemented with 20 ng/mL epidermal growth factor (EGF, Peprotech) and 40 ng/mL basal fibroblast growth factor (bFGF, Peprotech) and penicillin/streptomycin (P/S). Cells were maintained at 37 °C in a 5% CO_2_ incubator, and the medium was changed every 2–3 days. Cells at passage two were used in the experiments. Supporting the HFSC phenotype, the expression of the follicular stem cell marker cytokeratin 19 (K19) was verified by immunofluorescence staining. Briefly, P2 HFSCs were seeded on coverslips, fixed with 4% paraformaldehyde, permeabilized with 0.1% Triton X-100, and blocked with 5% BSA. The cells were then incubated with an anti-K19 primary antibody (1:200; Abcam) overnight at 4 °C, followed by incubation with an appropriate fluorescent secondary antibody and DAPI (1:500, Sigma) for nuclear counterstaining. The cells exhibited positive cytoplasmic staining for K19 under a fluorescence microscope, consistent with the reported phenotype of hair follicle stem cells (Li J. et al., 2022).

Normal human scalp hair follicles were obtained from three independent donors during hair transplantation procedures, and all follicles were in the anagen phase (n = 3). DPCs were isolated and expanded as described previously (Xiao et al., 2020). Briefly, follicle bulbs were transected from the same scalp hair follicle specimens obtained during hair transplantation procedures, and DP were microdissected from the bulbs and transferred to cell culture in DMEM supplemented with 10% (v/v) FBS and 1% (v/v) penicillin-streptomycin (100 U/mL penicillin and 100 μg/mL streptomycin). After 1 week of culture at 37 °C with 5% CO_2_, the DPCs were treated with 0.25% (wt/vol) trypsin-EDTA (Gibco) and split into two equal volumes for subculture. DPCs at passage three were used in subsequent experiments. DPC identity was confirmed by ALP and NCAM staining.

### 2.3 Preparation and characterization of hAAM

The hAAM used in this study was prepared from amniotic membranes obtained from three different healthy donors (n = 3). hAAM was prepared using a modified protocol based on previously published methods (Xiao et al., 2021). In brief, the HAM was separated from the chorion, frozen at −80 °C for 48 h, and then thawed prior to decellularization. To prepare the hAAM, the amniotic tissue was thoroughly washed with phosphate-buffered saline (PBS) to remove blood and cellular debris, followed by decellularization in a 1% Triton X-100 solution at 20 °C for 24 h. The membranes were then incubated in a 0.125% trypsin solution at 20 °C for 2 h, with the trypsin solution being changed after 1 hour. After digestion, the epithelium and cell debris were scraped off using a cell scraper. The hAAM was then subjected to repeated washing in PBS and dialyzed against double-distilled water at 4 °C for 24 h to remove excess reagents. Both intact hAM and hAAM were fixed in 4% paraformaldehyde (Solarbio) for further analysis, including hematoxylin and eosin (H&E) staining, DAPI staining, DNA quantification, and agarose gel electrophoresis. It should be noted that only the portion of the tissue that was subsequently analyzed by these methods was fixed in paraformaldehyde. After dialysis, decellularized hAAM was lyophilized overnight and subsequently sterilized overnight with ethylene oxide gas then stored at 4 °C. The surface morphology and structure of the hAAM scaffold were evaluated by scanning electron microscopy (SEM, Hitachi, Tokyo, Japan; S4800).

### 2.4 Compatibility assay of HFSCs and DPCs with hAAM and construction of cell-loaded composite grafts

This study first evaluated the *in vitro* biocompatibility and growth characteristics of HFSCs and DPCs on a decellularized hAAM scaffold. The three donor-derived hAAM batches and DPC isolates were pooled prior to use to ensure material consistency across all experimental groups; the HFSCs were obtained as a standardized commercial pooled batch from the supplier. The hAAM was then evenly placed in 24-well cell culture plates. HFSCs at passage 2 (digested with low-concentration trypsin) and P3 DPCs (digested with trypsin) were collected by centrifugation and seeded onto the hAAM at a density of 1 × 10^5^ cells/cm^2^. The cells were cultured in HFSC-specific medium (DELF primary keratinocyte culture medium supplemented with 20 ng/mL bFGF) or complete medium (DMEM/F12 with 10% FBS, penicillin/streptomycin, and EGF), and maintained at 37 °C in a 5% CO_2_ incubator. After 48 h of culture, cell growth and morphology were observed under an optical microscope. Samples were fixed for further examination by SEM to assess cell adhesion and spreading on the scaffold.

Based on the suggested biocompatibility of hAAM, composite grafts loaded with different cell combinations were further constructed. The hAAM was cut into circular discs with a diameter of 1 cm, sterilized, and pre-treated following the same procedure described above, then placed in 48-well plates. Using P2 HFSCs and P3 DPCs, three groups were prepared: 5 × 10^5^ cells HFSCs alone, 1 × 10^6^ cells DPCs alone, and a co-culture group containing both 5 × 10^5^ cells HFSCs and 1 × 10^6^ cells DPCs. Cells were seeded onto the hAAM surface, supplemented with complete medium consisting of DMEM/F12 (1:1) containing 10% FBS, 100 U/mL penicillin, 100 μg/mL streptomycin, and 10 ng/mL epidermal growth factor (EGF). For HFSC-specific medium, we used DELF primary keratinocyte culture medium (Wanwu Biotechnology, Hefei, China) supplemented with 20 ng/mL bFGF according to the supplier’s recommendation for HFSC expansion, rather than the complete medium used for DPCs and co-cultures. This process successfully produced hAAM grafts loaded with either single cell types or a co-culture system.

### 2.5 Evaluation of repair of full thickness skin defects in nude mice

BALB/c nude mice (n = 50 mice) were anesthetized with 2% isoflurane via inhalation at a flow rate of 0.41 mL/min. After anesthesia and skin preparation, a circular full-thickness excisional wound with a diameter of 1 cm was created on the dorsal skin of each mouse using a sterile biopsy punch and surgical scissors. The wound extended through the epidermis and dermis to the panniculus carnosus layer. After wound creation, mice were randomly assigned to five experimental groups using a random number table. Control group: the wound was covered with a gauze circle (10 mm in diameter) (n = 10); hAAM group: the wound was covered by a circular hAAM patch (10 mm in diameter) (n = 10); hAAM + HFSCs group (5 × 10^5^): the wound was covered by a circular hAAM patch (10 mm in diameter) combination of HFSCs (n = 10). hAAM + DPCs group (1 × 10^6^): the wound was covered by a circular hAAM patch (10 mm in diameter) combination of DPCs (n = 10). HAAM + HFSCs (5 × 10^5^) + DPCs group (1 × 10^6^): the wound was covered by a circular hAAM patch (10 mm in diameter) combination of HFSCs and DPCs. Animals were scheduled for tissue collection at postoperative days 7, 14, and 21, the entire wound bed together with approximately 2–3 mm of surrounding normal skin was excised. For each group, three animals were allocated to each time point (total n = 9 per group). The collected tissues were fixed in 4% paraformaldehyde, embedded in paraffin, and sectioned for histological and immunofluorescence analyses. One additional animal per group was maintained as a reserve animal to compensate for potential mortality or experimental loss. As no replacement was required during the study, the reserve animals were not included in the final statistical analyses. Only the data relevant to the objectives of the present study are presented in this manuscript.

After the surgery, the wounds were treated as indicated and then covered by Vaseline. Finally, TegadermTM (3 M, St. Paul, MN, United States) was used to fix the wound and dressings. The wound dressings in each group were changed every other day, three times in total according to the above methods. The mice were housed individually and wound healing was evaluated on the basis of gross observation at days 3, 5,7, 10 and 14 post-operation. A fixed cohort of three animals per group was continuously tracked from day 3 to day 14 for wound closure assessment. These same three animals were subsequently used for tissue collection at day 14. An additional three animals per group were used exclusively for tissue collection at day 7, and the remaining three animals per group were used exclusively for tissue collection at day 21. The 10th animal in each group served as a reserve to compensate for potential mortality or experimental loss; as no replacement was required during the study, the reserve animals were not included in the final statistical analyses. Thus, tissue samples were collected from three animals per group at each time point (days 7, 14, and 21), and the longitudinal wound closure analysis was based on the same cohort of three animals per group (n = 3 per time point, including day 14). Wound images were captured with a digital camera, and the wound area was measured using Image software (National Institutes of Health, United States) by tracing the wound margins. To ensure accuracy and minimize bias, measurements were performed independently by two investigators who were blinded to the treatment groups; the average of the two measurements was used for statistical analysis. The wound healing rate was calculated using the following formula: Wound healing rate = [(Initial wound area - Residual wound area)/Initial wound area] × 100%.

### 2.6 Histological analysis

Wound areas, including surrounding skin, were collected at 14 and 21 days post-operation. Samples were fixed in 4% paraformaldehyde (Solarbio), gradually dehydrated, embedded in paraffin, and cut into 4 μm sections. The sections were stained with H&E or Masson’s trichrome stain according to the manufacturer’s instructions (Sigma-Aldrich). H&E staining was used to evaluate epidermal thickness and the presence of morphological structures suggestive of skin appendages, including hair follicles and sebaceous glands, while Masson’s staining was used to determine the content and maturity of collagen in the wound beds. Image-Pro Plus 6.0 software was used to calculate the collagen deposition at days 14 post-operation. For histological and immunofluorescence analyses, five random high-power fields per sample were evaluated. The data from these five fields were first averaged within each animal to generate a single representative value (n = 3 animals per group). All statistical analyses were performed based on these biological replicates rather than individual microscopic fields.

### 2.7 Immunofluorescence staining

The wound tissues were used for immunofluorescence staining. For immunofluorescence staining, paraffin-embedded sections were deparaffinized and rehydrated. Heat-induced antigen retrieval was performed using citrate buffer (pH 6.0) or Tris-EDTA buffer (pH 9.0) in a microwave oven for 30 min. After cooling, sections were blocked with 3% bovine serum albumin (BSA) or 10% normal donkey serum for 30 min at room temperature. The sections were then incubated with the following primary antibodies overnight at 4 °C: anti-CD31 (GB113151, 1:500, Servicebio), anti-Ki67 (GB111141, 1:600, Servicebio), anti-Collagen I (GB115707, 1:1000, Servicebio), anti-Collagen III (GB111629, 1:500, Servicebio), anti-BMP-4 (GB11520, 1:2000, Servicebio), anti-α-SMA (GB12045, 1:1000, Servicebio), anti-TGF-β (81746-RR, 1:1000, Proteintech), anti-human nucleoli (HNA, ab191181, 1:500, Abcam), and anti-PPAR-γ (16643-1-AP, 1:500, Proteintech). After washing with PBS, the sections for CD31, Ki67, Col I, Col III, BMP-4, TGF-β, and α-SMA were incubated with CY3-conjugated goat anti-rabbit IgG (GB21303, 1:300, Servicebio) or CY3-conjugated goat anti-mouse IgG (GB21301, 1:300, Servicebio) for 50 min at room temperature in the dark. For HNA and PPAR-γ staining, a Tyramide Signal Amplification (TSA) fluorescence kit (D1002plus-01RMPlus, Zhongkang Biotech) was applied following the manufacturer’s instructions to enhance the signal. Nuclei were counterstained with DAPI. Finally, sections were mounted with an anti-fade mounting medium. The fluorescence intensity of CD31, Ki67, Col I, Col III, BMP-4, TGF-β, and α-SMA was quantified using ImageJ software. Images were acquired under identical exposure settings, and relative fluorescence intensity (RFI) was measured within standardized regions of interest (ROIs). Fluorescence values were normalized to the analyzed image area and used for comparison among groups.

### 2.8 Euthanasia protocol

In this study, all mice were humanely euthanized in compliance with institutional animal care protocols and the AVMA Guidelines for the Euthanasia of Animals. Deep anesthesia was induced using inhaled isoflurane (5% for induction in an induction chamber, followed by 1%–2% for maintenance via nose cone in 100% oxygen) delivered through a precision vaporizer. Upon achieving a surgical plane of anesthesia, indicated by the absence of reflexes, tissue samples were collected as required. Cervical dislocation was then performed as a secondary physical method to ensure death while animals remained fully anesthetized.

### 2.9 Statistical analysis

Data were tested for normality using the Shapiro-Wilk test and for homogeneity of variance using Levene’s test. For normally distributed data with equal variances, comparisons between multiple groups were performed using one-way analysis of variance (ANOVA) followed by Tukey’s *post hoc* test to correct for multiple comparisons. For the longitudinal wound closure analysis (Days 3–14), repeated measurements were obtained from a fixed cohort of the same animals over time. These data were analyzed using a repeated-measures analysis of variance (ANOVA), with Time as the within-subject factor and Treatment group as the between-subject factor; the Greenhouse-Geisser correction was applied when sphericity was violated. This was followed by Bonferroni’s *post hoc* test for multiple comparisons. The analysis was performed using the General Linear Model (GLM) Repeated Measures procedure in SPSS 29.0 (IBM Corp., Armonk, NY, United States). A two-tailed P-value of <0.05 was considered statistically significant. All image acquisition, histological quantification, and statistical analyses were performed by investigators blinded to the treatment allocation.

## 3 Results

### 3.1 Characterization of DPCs and HFSCs

Under light microscopy, HFSCs displayed a paving stone-like cellular arrangement, and immunofluorescence suggested positive expression of the HFSC surface marker K19 (Figures 1A,B). DPCs were successfully isolated and cultured, with cells migrating out from the dermal papilla tissue within 24 h (Figure 1C); P1 DPCs exhibited a typical spindle-shaped morphology (Figure 1D). DPCs demonstrated strong NCAM expression and ALP activity, and immunofluorescence further suggested strong positive expression of ALP and NCAM in the isolated DPCs (Figures 1E,F). Collectively, these results suggest that purchased HFSCs and isolated DPCs display the characteristic features of their respective cell types.

**FIGURE 1:**
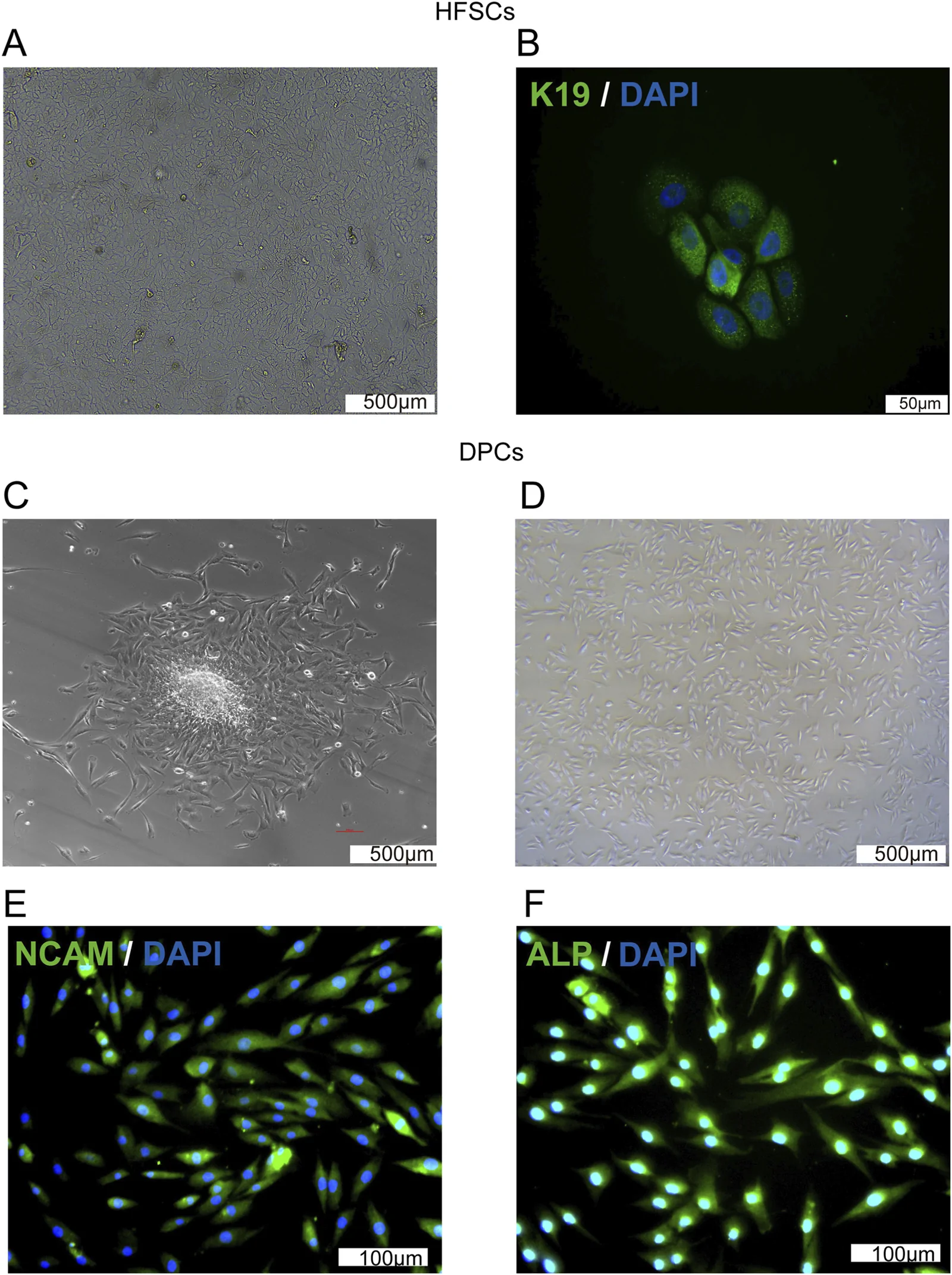
Isolation, culture, and characterization of human hair follicle stem cells and human dermal papilla cells. **(A)** Morphology of primary human HFSCs (scale bar: 500 μm). **(B)** Immunofluorescence staining of HFSCs marker: representative positive expression of K19 (green); nuclei were counterstained with DAPI (blue) (scale bar: 50 μm). **(C)** Representative area showing early cell outgrowth from dermal papilla explants 24 h after seeding. (scale bar: 500 μm). **(D)** Morphology of primary human DPCs at passage 1 (scale bar: 500 μm). **(E,F)** Immunofluorescence staining of DPC markers: representative positive expression of NCAM (green) and ALP (green); nuclei were counterstained with DAPI (blue) (scale bar: 100 μm).

### 3.2 Characterization of hAAM

The matrix structures of native hAM and hAAM were visualized by H&E staining and almost no residual cells were observed (Figures 2A,B). DAPI staining revealed a significant presence of blue fluorescent nuclei in hAM, while hAAM showed almost no blue fluorescent nuclei (Figures 2C,D). The morphology of hAM before and after decellularization was observed by SEM. The epithelial cells of hAM arranged in a flat paving stone-like appearance (Figure 2E). After decellularization, the hAAM revealed a three-dimensional porous structure arranged by collagen fibers, with many cell niches, and no obvious epithelial cells remained (Figure 2F). Agarose gel electrophoresis suggested that hAAM contained no DNA fragment larger than 200 bp, while there were plenty of them in hAM (Figure 2G) (The 500 bp marker was highlighted for reference purposes). The total DNA content of hAAM was measured at 0.005 μg/mg, significantly lower than that of hAM, which was 0.43 μg/mg, based on measurements from three independent donor preparations (n = 3; Figure 2H). These results collectively suggest that hAAM underwent effective decellularization. In addition, Microscopic observation revealed that both human hair follicle stem cells and human dermal papilla cells, when seeded on human acellular amniotic membrane under both monoculture and coculture conditions, adhered and expanded favorably. To evaluate the supporting role of hAAM for hair follicle-derived cells, we further observed the two-dimensional growth morphology of cells on the material using light microscopy. The results showed that both individually cultured HFSCs and DPCs, as well as their co-culture, adhered and spread well on the surface of hAAM, exhibiting typical proliferative cell morphology (Figures 2I–K). SEM observations demonstrated that both HFSCs and DPCs successfully adhered to the hAAM surface and exhibited a spread morphology with close contact to the scaffold. (Figures 2L,M), demonstrating favorable compatibility with the scaffold material and supporting cell attachment and growth.

**FIGURE 2:**
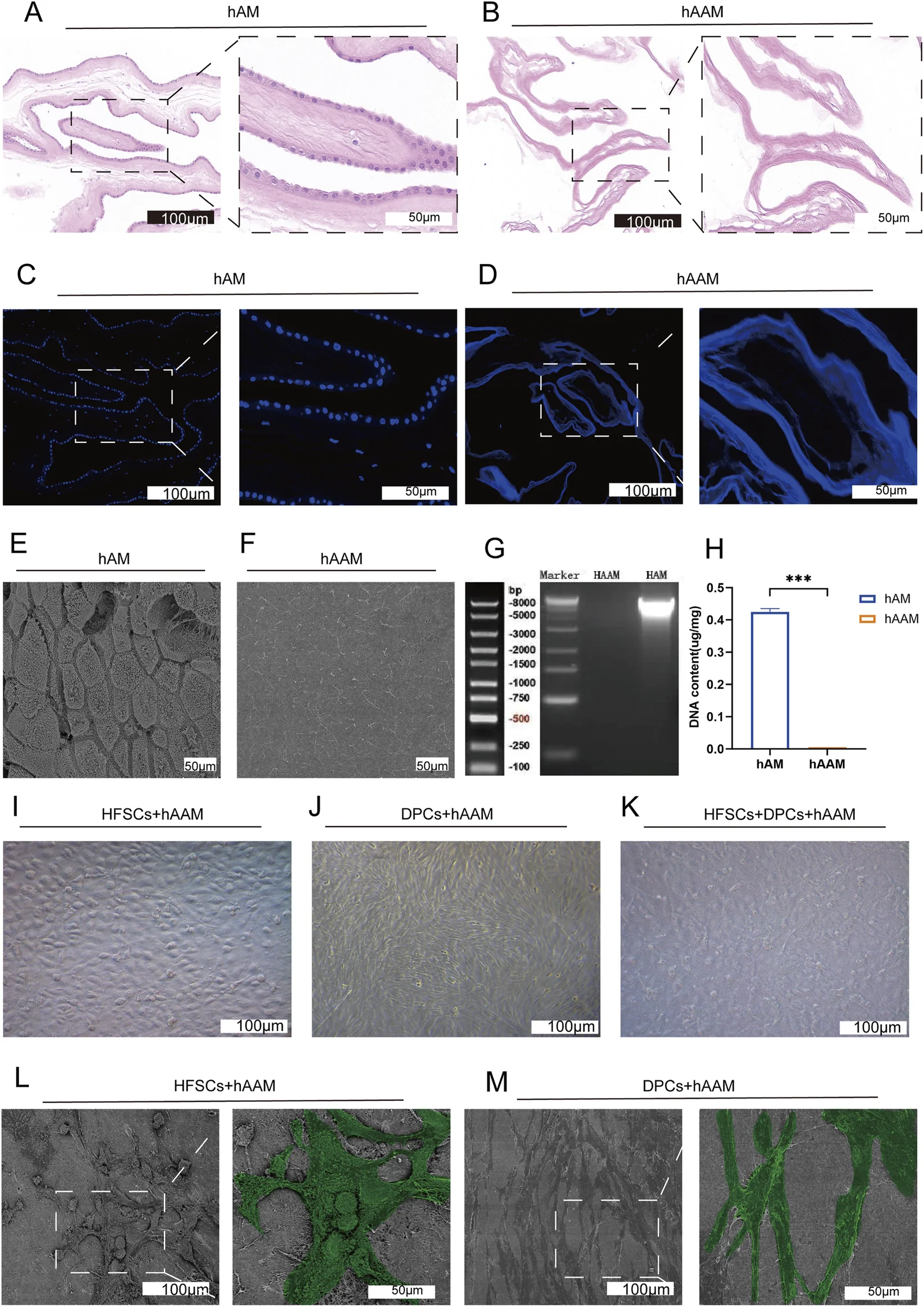
Characterization of hAAM. **(A)** Hematoxylin and eosin (H and E) staining of hAM before decellularization, showing the presence of a large number of nuclei (scale bar = 100 μm). **(B)** H&E staining of hAM after decellularization showing almost no cell residue (scale bar = 100 μm). **(C)** DAPI staining indicates a substantial number of nuclei in hAM, displaying blue fluorescence (scale = 100 μm). **(D)** DAPI staining reveals a minimal number of nuclei in hAAM, showing blue fluorescence (scale = 100 μm). **(E)** Microstructures of the hAM before decellularization under SEM showed that epithelial cells arranged in a flat paving stone-like appearance on the epithelial side (scale bar = 50 μm). **(F)** Microstructures of the hAAM after decellularization under SEM showed the epithelial cells were removed after decellularization, and cell niches were observed on the epithelia side of the hAAM (scale bar = 50 μm). **(G)** Agarose gel electrophoresis results for hAM and hAAM. **(H)** Quantification of total DNA content in hAM and hAAM. Data are presented as mean ± SD from three independent donor preparations (n = 3). **(I)** Light microscopy shows that HFSCs adhered and proliferated on the hAAM, exhibiting a cobblestone-like morphology with visible cellular extensions and intercellular connections, eventually forming confluent cell sheets (scale bars:100 μm). **(J)** Light microscopy shows that DPCs adhered and proliferated on the hAAM, displaying a flat spindle-shaped morphology and forming interconnected cell sheets (scale bars:100 μm). **(K)** Light microscopy observation shows that at 24 h post-seeding, the co-cultured DPCs and HFSCs adhered to the hAAM, with partial cells aggregating into clusters (scale bars:100 μm). **(L,M)** SEM analysis shows that HFSCs (L) and DPCs (M) seeded on the hAAM exhibit a flaky, clustered growth pattern, with some cells occupying the cell niches on the membrane surface. The attached cells in the magnified SEM images were pseudocolored in green to improve visualization of cell morphology and distribution on the scaffold surface. (scale bars 100 μm). *P < 0.05, **P < 0.01, ***P < 0.001, ****P < 0.0001.

### 3.3 hAAM + HFSCs + DPCs accelerates nude full-thickness back skin wound healing

The efficacy of hAAM + HFSCs + DPCs for wound healing was investigated in nude mice. As shown in Figures 3A–C, the hAAM + HFSCs + DPCs group exhibited significantly accelerated wound closure compared with the other groups at day 14 (Bonferroni *post hoc* test: *P* < 0.001 vs. Control; *P* < 0.001 vs. hAAM; *P* < 0.01 vs. hAAM + HFSCs and hAAM + DPCs). Repeated-measures ANOVA revealed a significant overall group effect (*F* (Zhou et al., 2021; Farabi et al., 2024) = 114.159, *P* < 0.001) and a significant Time × Group interaction (*F* (8.125, 20.312) = 4.611, *P* = 0.002; Greenhouse–Geisser corrected), indicating that the healing trajectories differed significantly among the five groups over the 14-day observation period (Supplementary Table S1). To decouple wound contraction from true epithelial regeneration, we further quantified the wound length on day 7 and the scar length on day 14 using histology (Supplementary Figure S1). On day 7, the hAAM + HFSCs + DPCs group exhibited a significantly shortened wound length compared with the other groups (Supplementary Figure S1A and B), suggesting accelerated early epithelial migration. By day 14, although macroscopic wound closure was largely driven by contraction, all groups exhibited complete epidermal closure (Supplementary Figure S1C). Measurement of the residual scar length (Supplementary Figure S1D) revealed that the hAAM + HFSCs + DPCs group had the smallest scar size, reflecting enhanced wound contraction. To further evaluate the therapeutic effects of hAAM + HFSCs + DPCs, regenerated wound tissue samples from the five groups were collected on day 14, day 21 for histological analysis, including hematoxylin and eosin (H&E) staining and Masson staining. The histopathological evaluation focused on wound healing parameters, including epidermal thickness, and collagen fraction (Han et al., 2024; Wu et al., 2023). Wounds treated with hAAM + HFSCs + DPCs exhibited the thickest epidermal layer among all groups (Figures 3D,E), which we interpret as an active hyperplastic response rather than a definitive mature skin structure. At day 21, glandular formations resembling potential sebaceous gland-like structures were observed in the hAAM + DPCs and hAAM + HFSCs + DPCs groups based on H&E morphology (Figure 3D). In the hAAM + HFSCs + DPCs group, Masson staining revealed increased collagen deposition with a more homogeneous distribution compared with the other groups (Figures 3F,G).

**FIGURE 3:**
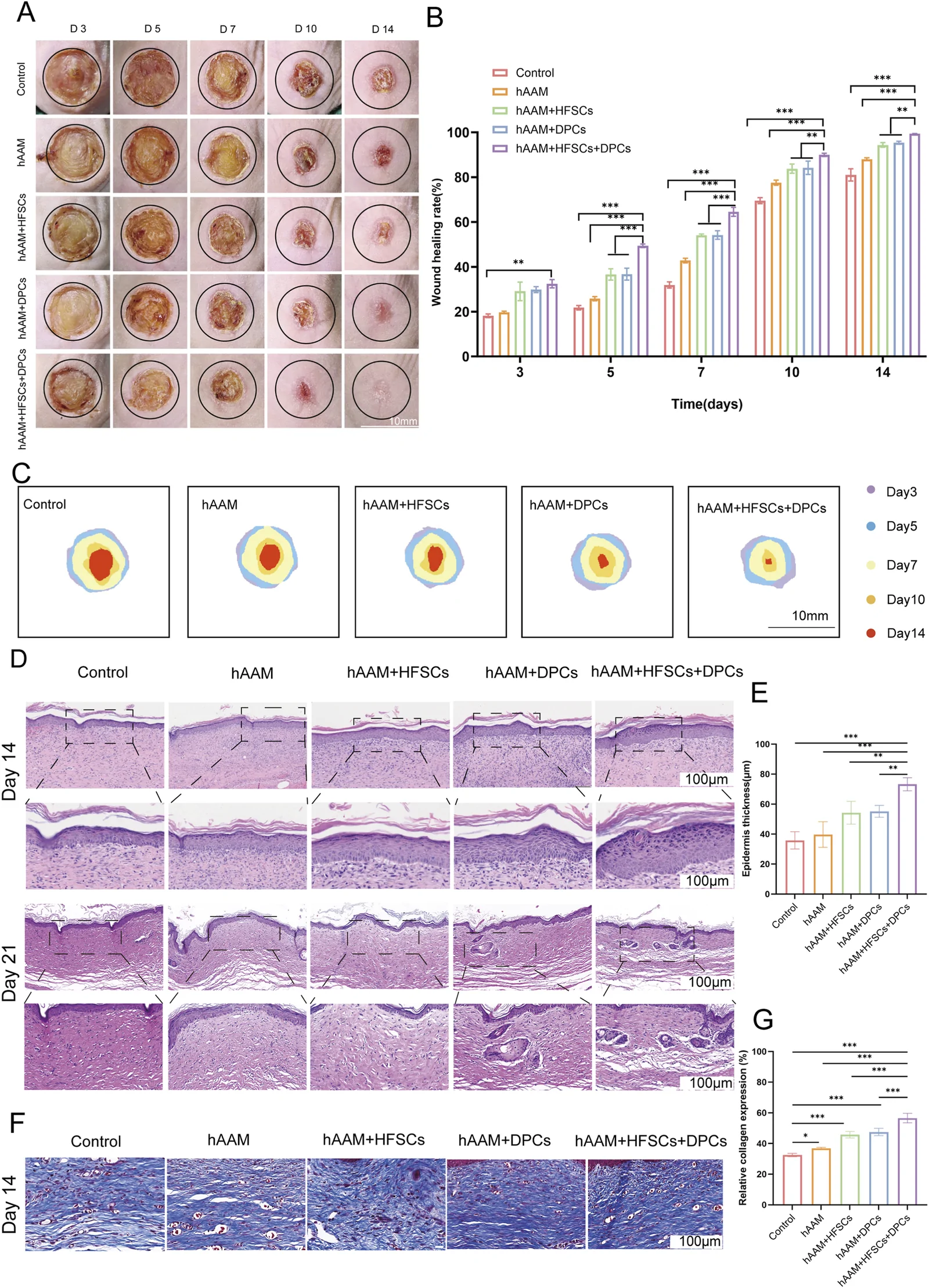
The hAAM + HFSCs + DPCs composite scaffold promotes wound healing on the dorsal skin of nude mice. **(A)** Representative macroscopic images of wound healing in mice from different treatment groups. **(B)** Wound closure rates across five groups at different time points. Data are presented as mean ± SD. Longitudinal measurements were obtained from a fixed cohort of n = 3 animals per group at all time points. **(C)** Wound closure trajectory from day 3 to day 14. **(D)** H&E staining of wound tissues on day 14 and day 21 (scale bar: 100 μm). **(E)** Quantitative analysis of epidermal thickness. **(F,G)** Masson staining and corresponding collagen volume fraction quantification of wound tissues on day 14 (scale bar: 100 μm). Data are presented as mean ± SD (n = 3 animals per group, postoperative day 14). For longitudinal data in **(B)**, statistical significance was determined by repeated-measures ANOVA with Greenhouse–Geisser correction followed by Bonferroni’s *post hoc* test. The comparison bars and symbols in **(B)** denote significant differences between the hAAM + HFSCs + DPCs group and each of the other four groups (Control, hAAM, hAAM + HFSCs, and hAAM + DPCs) at the corresponding time points. For single time-point data in **(E,G)**, statistical significance was determined by one-way ANOVA followed by Tukey’s *post hoc* test. In **(E)**, comparison bars and symbols denote significant differences between the hAAM + HFSCs + DPCs group and each of the other four groups. In **(G)**, comparison bars and symbols denote the following pairwise comparisons: hAAM + HFSCs + DPCs vs. Control (***), vs. hAAM (***), vs. hAAM + HFSCs (***), and vs. hAAM + DPCs (***); hAAM + DPCs vs. Control (***). hAAM + HFSCs vs. Control (***). *P < 0.05, **P < 0.01, ***P < 0.001, ****P < 0.0001.

### 3.4 hAAM + HFSCs + DPCs modulates the proliferation and remodeling phases of wound healing in a nude mouse full-thickness wound model

The proliferative phase is the second stage of wound repair, distinguished by local neovascularization and cellular proliferation (Gurtner et al., 2008). We used immunofluorescence staining for CD31 (a transmembrane protein expressed in the early stage of vascular growth) to assess presence of CD31-positive vascular structures. After 14 days of treatment, the hAAM + HFSCs + DPCs group revealed higher CD31 fluorescence intensity and a greater number of CD31-positive vascular structures compared with the control group (Figures 4A,B). To further quantify this observation, we measured CD31^+^ vessel density (vascular profiles per mm^2^) based on the immunofluorescence images. As shown in Supplementary Figure S2, the hAAM + HFSCs + DPCs group exhibited a significantly higher vessel density compared with the other groups (*P* < 0.001). Moreover, the expression of a cell proliferation marker (Ki67) substantially increased following hAAM + HFSCs + DPCs treatment, as determined via immunofluorescence staining (Figures 4A,C). Taken together, these results demonstrated that the hAAM + HFSCs + DPCs group exhibited increased density of CD31-positive vascular structures and increased Ki67-positive cell proliferation at postoperative day 14 compared with the other treatment groups.

**FIGURE 4:**
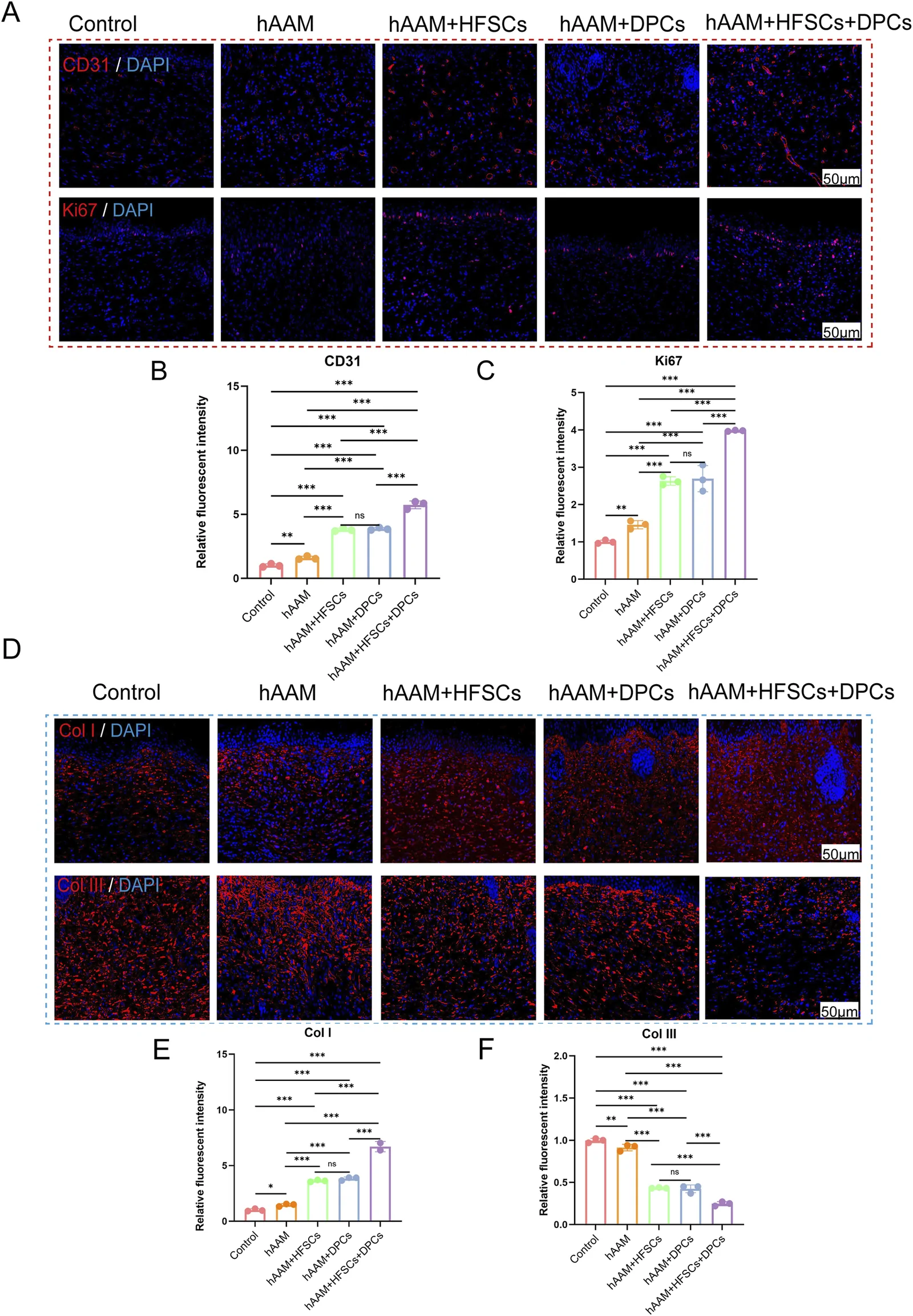
Immunofluorescence analysis of CD31-positive vascular structures, Ki67-positive cell proliferation, and collagen deposition during wound healing on postoperative day 14. **(A)** Representative immunofluorescence images showing staining for CD31, Ki67. Scale bar: 50 μm. **(B,C)** Quantitative analysis of relative fluorescence intensity (RFI) normalized to the analyzed image area for **(B)** CD31, **(C)** Ki67. **(D)** Representative immunofluorescence images showing staining for Col I, Col III. Scale bar: 50 μm. **(E,F)** Quantitative analysis of relative fluorescence intensity (RFI) normalized to the analyzed image area for **(E)** Col I, **(F)** Col III. Data are presented as mean ± SD (n = 3 animals per group, postoperative day 14). *P < 0.05, **P < 0.01, ***P < 0.001, ****P < 0.0001.

The last stage of wound repair is the remodeling phase, a process involving collagen deposition and maturation (Wang et al., 2025). In this phase, Collagen type III (Col III), with low tensile strength and high elasticity, dominates the provisional ECM and forms a disorganized fibrillar network, whereas collagen type I (Col I), with high tensile strength and high toughness, progressively replaces Col III and drives ECM maturation in the remodeling phase (Widgerow, 2011). In this study, we performed immunofluorescence staining for collagens in newly formed skin tissue on day 14 (Figures 4D–F). Compared with the control group, increased collagen I expression and decreased collagen III expression were observed in the hAAM + HFSCs + DPCs group. To further assess the balance of collagen remodeling, the Col I/Col III ratio was calculated (Supplementary Figure S3). The hAAM + HFSCs + DPCs group exhibited an altered Col I/Col III ratio compared with the other groups. This altered Col I/Col III ratio at day 14 was accompanied by reduced immunostaining of fibrosis-related markers, demonstrating an altered remodeling profile. However, definitive conclusions regarding matrix maturation or long-term fibrosis reduction cannot be drawn from these current data.

### 3.5 Altered expression of fibrosis-associated markers in the hAAM + HFSCs + DPCs group

Research indicates that the bone morphogenetic protein (BMP) signaling pathway can induce adipogenic commitment of cells *in vitro* (Gupta et al., 2010; Hammarstedt et al., 2013). BMP is expressed in HFSCs, DPCs, inner root sheath cells, and outer root sheath cells. Furthermore, actively growing hair follicles, which are critical for myofibroblast-to-adipocyte reprogramming, strongly express BMP2 and BMP-4 (Plikus et al., 2017). Further studies have shown that BMP is involved in the repair process following tissue injury and can mitigate TGF-βinduced fibrosis in multiple organs (Dituri et al., 2019). Thus, BMP expressed by hair follicle-derived cells may be associated with the modulation of fibrotic responses during wound healing. To evaluate tissue remodeling and fibrosis during wound healing, immunofluorescence staining for BMP-4, TGF-β, and α-SMA was performed on wound tissues harvested on postoperative day 14. As shown in Figure 5, compared with the various control groups, the hAAM + HFSCs + DPCs treatment group exhibited a significant upregulation in BMP-4 protein expression (Figures 5A,B), which was visually predominantly distributed in the superficial epidermal regions. Conversely, the expression of TGF-β, a key profibrotic factor, was the lowest in this group, showing a trend negatively correlated with BMP-4 expression (Figures 5A,C). Furthermore, the positive area and staining intensity for α-SMA were significantly lower in the hAAM + HFSCs + DPCs group than in all other groups. Given that α-SMA can be expressed by multiple cell types, including myofibroblasts, vascular smooth muscle cells, and pericytes, these findings suggest altered tissue remodeling but do not definitively establish changes in fibrotic activity. (Figures 5A,D). Collectively, the increased BMP-4 expression, together with reduced TGF-β and α-SMA immunostaining, demonstrates an association with altered tissue remodeling and reduced immunostaining of fibrosis-associated markers in the hAAM + HFSCs + DPCs group. However, these findings establish a correlation rather than a causal mechanism, as the cellular source of BMP-4 and its direct role in modulating fibrosis-associated markers remain to be determined.

**FIGURE 5:**
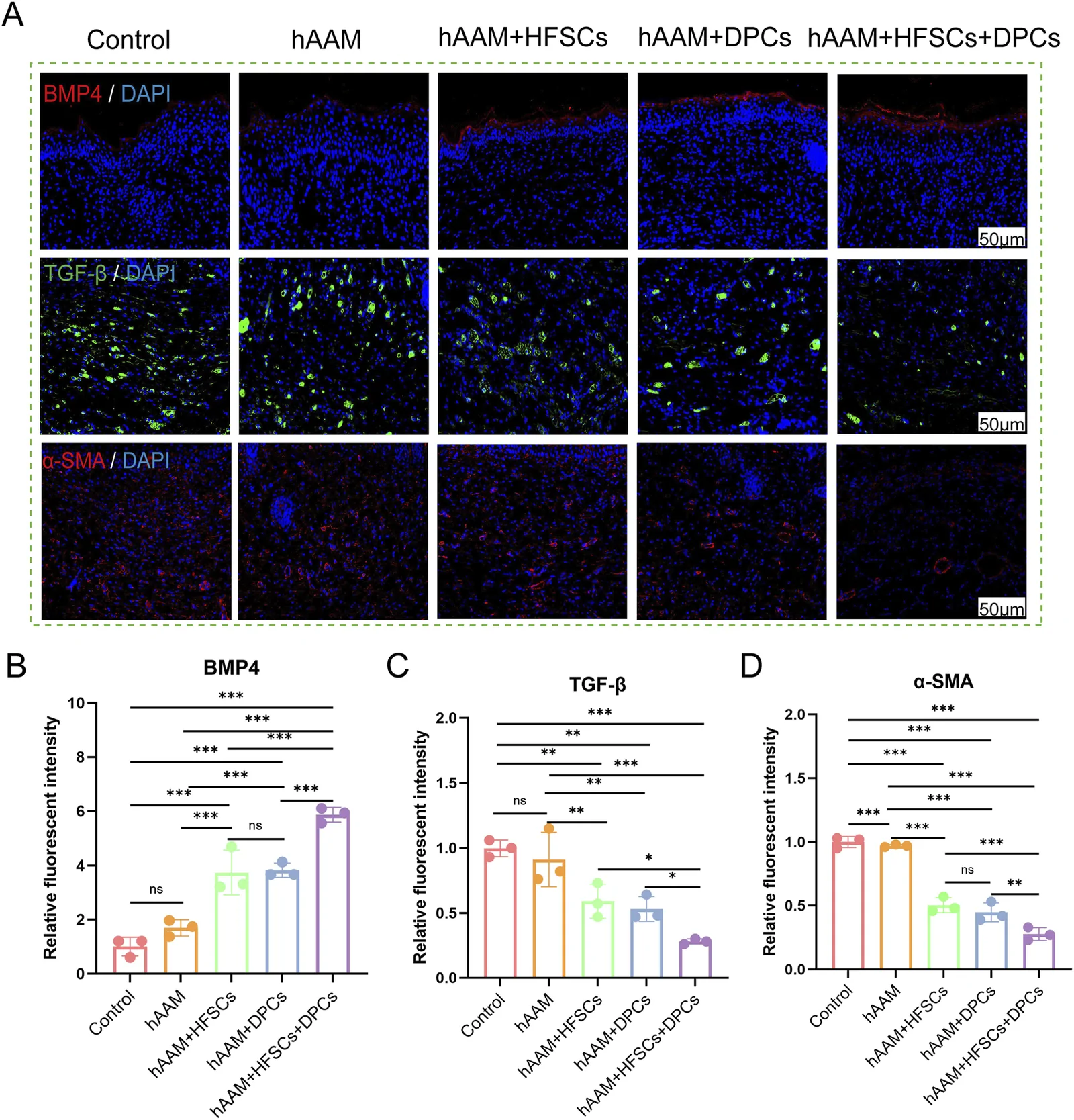
Immunofluorescence analysis of fibrosis-related markers in wound healing on postoperative day 14. **(A)** Representative immunofluorescence images showing staining for BMP-4, TGF-β and α-SMA in wound sections. Scale bar: 50 μm. **(B–D)** Quantitative analysis of relative fluorescence intensity (RFI) normalized to the analyzed image area for **(B)** BMP-4, **(C)** TGF-β, and **(D)** α-SMA. Data are presented as mean ± SD (n = 3 animals per group, postoperative day 14). *P < 0.05, **P < 0.01, ***P < 0.001, ****P < 0.0001.

### 3.6 Observation of appendage-like structures following hAAM-mediated delivery of HFSCs and DPCs

To evaluate HNA immunoreactivity in the wound area and to further characterize the appendage-like structures observed during wound repair, immunofluorescence analysis was performed on tissue samples collected at different postoperative time points. On day 14, anti-human nucleoli antibody staining revealed positive signals within the wound area in groups receiving human DPCs and/or HFSCs. Although a certain degree of non-specific background staining was observed in the control and hAAM groups, the HNA-positive signals were relatively more abundant in the cell-transplanted groups, predominantly localized in the dermal region. However, due to incomplete nuclear co-localization with DAPI, these findings should be described as HNA immunoreactivity of uncertain specificity (Figure 6A), rather than definitive evidence of transplanted human-cell persistence. Subsequently, cells positive for PPAR-γ were detected within structures that morphologically resembled potential sebaceous glands in the hAAM + HFSCs + DPCs and hAAM + DPCs groups (Figure 6B). To quantify their incidence, a semi-quantitative analysis was performed. As summarized in Supplementary Table S2, in the hAAM + HFSCs + DPCs group, both structure types were present in all available specimens (occurrence rate: 3/3, 100%), with 2-4 PPAR-γ-positive gland-like structures and 1–7 epithelial bud-like structures morphologically suggestive of early follicular structures per wound. In contrast, the hAAM + DPCs group had a lower occurrence rate (1/3, 33.3%), presenting 1 PPAR-γ-positive gland-like structures and 1 epithelial bud-like structures morphologically suggestive of early follicular structures in the positive specimen. However, based on limited immunophenotypic characterization, we cannot definitively exclude the possibility that these structures represent non-specific epithelial aggregates.

**FIGURE 6:**
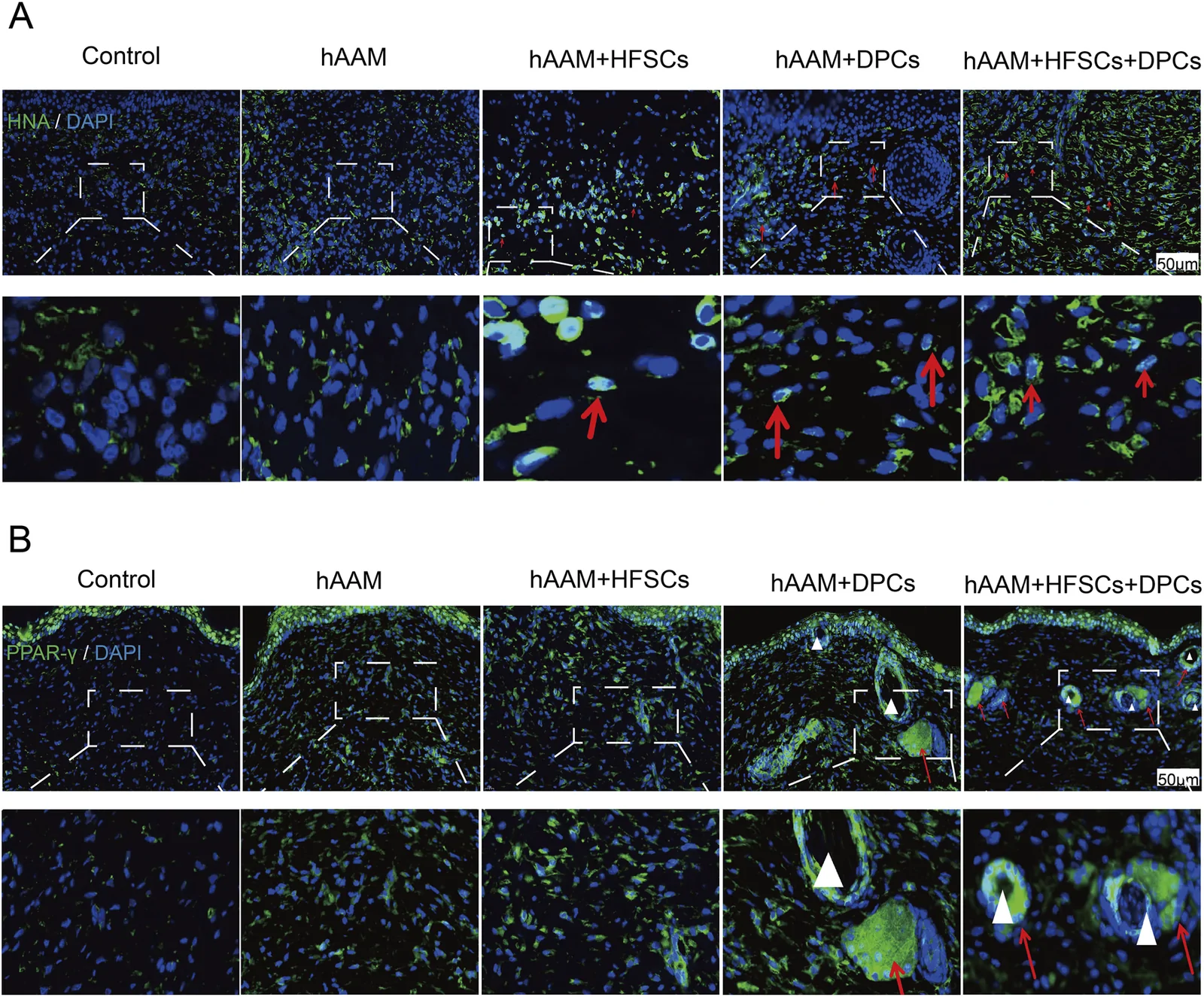
Assessment of HNA immunoreactivity and characterization of structures with appendage-like morphology. **(A)** Anti-human nucleoli antibody staining on postoperative day 14. Signals (red arrowheads) were detected within the dermis; however, due to non-specific background staining and incomplete co-localization with DAPI (blue), these signals are described as HNA immunoreactivity of uncertain specificity. Scale bar: 50 μm. **(B)** PPAR-γ staining on postoperative day 21. PPAR-γ-positive structures with morphological features resembling sebaceous glands (red arrowheads) and epithelial bud-like structures morphologically suggestive of early follicular development (white arrowheads) were observed in the hAAM + HFSCs + DPCs group. Scale bar: 50 μm. Immunophenotypic characterization of these structures was limited to PPAR-γ staining; additional lineage-specific markers were not assessed.

## 4 Discussion

Deep skin injuries heal with scar formation, which prevents the regeneration of skin appendages such as HF and glands. HF regeneration during wound healing is essential for restoring skin structure and function, representing an optimal healing approach and a hallmark of regenerative wound healing (Mascharak et al., 2022; Zheng et al., 2024). In this study, we developed an hAAM-based combinatorial strategy for co-delivery of HFSCs and DPCs and evaluated its effects in a murine full-thickness wound model. The hAAM + HFSCs + DPCs group showed improved gross wound closure, increased CD31 and Ki67 staining, altered extracellular matrix remodeling, and the appearance of appendage-like structures in some specimens. These findings suggest that the combined delivery of epithelial-like HFSCs and mesenchymal-like DPCs may improve wound repair compared with either cell type alone. However, the current data do not demonstrate functional skin regeneration, mature appendage formation, or direct structural contribution of transplanted human cells.

HF morphogenesis relies on the epithelial–mesenchymal interaction (EMI) (Zhang and Chen, 2024; Sennett and Rendl, 2012). With DPCs as an inducible mesenchymal component to drive HF formation. HFSCs possess strong proliferative and differentiation potential, contributing to rapid re-epithelialization and epidermal homeostasis (Peterson and Nair, 2022). Meanwhile, DPCs regulate dermal repair by secreting growth factors, promoting neovascularization, and directing epithelial cell fate (Madaan et al., 2018). In this study, wounds treated with both HFSCs and DPCs exhibited improved wound healing outcomes compared with wounds treated with either cell type alone, including increased epidermal thickness and more compact and homogeneous collagen deposition. These findings indicate that co-delivery of HFSCs and DPCs within the hAAM scaffold further improves wound healing, providing potential advantages for tissue repair.

The choice of scaffold material plays a pivotal role in stem-cell-based wound therapies. hAAM is an ideal biological scaffold due to its biocompatibility, low immunogenicity, natural ECM architecture, and abundance of collagen fibers (Li Y. et al., 2023; Xiao et al., 2021; Guo et al., 2024). In this study, the successful decellularization of human acellular amniotic membrane (hAAM) was suggested by H&E and DAPI staining, DNA quantification, and agarose gel electrophoresis. The results demonstrated minimal residual cellular content, a total DNA content below 50 ng/mg, and the absence of DNA fragments larger than 200 bp, meeting the criteria for acellular biological products (García-Gareta et al., 2020). SEM analysis revealed that hAAM retained a three-dimensional porous structure capable of supporting the adhesion and proliferation of HFSCs and DPCs, with cells forming intercellular connections, indicating that hAAM provides a favorable physical and biocompatible microenvironment. Moreover, hAAM exhibited excellent swelling and moisture-retention properties, facilitating the loading of stem cells or growth factors and providing a hydrated environment conducive to wound healing (Korting et al., 2011). Collectively, these findings suggest that the prepared hAAM not only meets the standards of an acellular biological scaffold but also demonstrates strong potential as a tissue engineering platform and as a delivery vehicle for cells or bioactive factors in regenerative wound therapy. Mechanical characterization of biomaterials is important to evaluate their suitability for tissue engineering applications. Previous studies have demonstrated that decellularized human amniotic membrane retains key extracellular matrix components such as collagen and elastin, thereby preserving its structural integrity and essential biomechanical properties (Bemenderfer et al., 2019; Parmar et al., 2025; Ramakrishnan et al., 2020). Moreover, in our study, histological analysis showed preservation of the native architecture after decellularization, and the scaffold effectively supported cell attachment and tissue regeneration *in vivo*. These findings indirectly suggest that the hAAM maintained sufficient structural integrity for wound repair applications. Nevertheless, comprehensive mechanical characterization will be necessary in future studies to further validate its biomechanical performance.

The hAAM + HFSCs + DPCs group exhibited increased CD31 and Ki67 expression compared with the other treatment groups, suggesting increased CD31-positive vascular structures and Ki67-positive cell proliferation within the healing tissue. These changes may contribute to the observed tissue repair; however, because we did not assess vascular perfusion or function, these findings should be interpreted as increased vascular structures rather than functional vascularization.

Changes in Col I and Col III staining suggested altered extracellular matrix remodeling in the treated wounds. Col I is a major structural component of mature skin and plays an important role in restoring tissue strength during wound repair. However, increased collagen I deposition alone should not be interpreted as unequivocal evidence of improved healing, as excessive collagen I accumulation may also be associated with fibrotic scar formation. Therefore, the biological significance of collagen I expression should be evaluated in conjunction with collagen III expression, collagen organization, and other fibrosis-related markers. In the present study, the observed changes in collagen I expression were interpreted together with Masson staining, collagen architecture, BMP-4, TGF-β, and α-SMA expression, collectively suggesting an altered remodeling profile during wound healing. However, definitive conclusions regarding matrix maturation or long-term fibrosis reduction cannot be drawn from these current data.

The hAAM + HFSCs + DPCs group showed increased BMP-4 staining accompanied by reduced TGF-β and α-SMA staining. These findings may indicate altered remodeling-associated signaling. The increased BMP-4 expression observed in the composite group may reflect altered morphogen signaling within the regenerating wound microenvironment. However, we acknowledge several critical limitations in the current study regarding the interpretation of these findings. First, visually, the BMP-4 immunofluorescence signal was predominantly distributed in the superficial epidermal regions rather than the dermal layer where transplanted human cells were primarily located. Therefore, we cannot definitively attribute the upregulated BMP-4 to the transplanted HFSCs or DPCs; it may be derived from host epidermal cells or represent a non-specific phenomenon associated with the keratinized layer. Second, given the lack of functional modulation experiments (such as BMP receptor blockade, pathway inhibition, or gene knockdown), our data strictly demonstrate a statistical association rather than a causal mechanism. Moreover, α-SMA is not specific to myofibroblasts and may also label vascular smooth muscle cells and pericytes. Therefore, the current data cannot establish a causal BMP-4-mediated anti-fibrotic mechanism or definitively quantify fibrosis-associated myofibroblasts. While previous studies have suggested that BMP-4 may exert anti-fibrotic effects by antagonizing TGF-β and reprogramming myofibroblasts into adipocytes (Plikus et al., 2017; Hoerst et al., 2019; Teng et al., 2022), our study merely identifies a correlation between increased BMP-4 immunoreactivity and reduced TGF-β/α-SMA expression. Consequently, the current findings should be interpreted as an association between local morphogen signaling shifts and altered fibrosis-associated markers, rather than direct evidence of a BMP-mediated mechanism. Future studies incorporating functional modulation of BMP signaling (e.g., BMP inhibitors or agonists), along with earlier time-point assessments and lineage tracing, will be necessary to determine the definitive role of this pathway. Additionally, it is important to emphasize that our observation period was limited to 21 days. While reduced expression of fibrosis-associated markers (TGF-β and α-SMA) and an altered Col I/Col III ratio were observed, these early intermediate-phase findings cannot be extrapolated to long-term scar prevention or permanent functional skin restoration. Extended observation periods and biomechanical assessments in appropriate animal models will be required to definitively establish whether this composite construct can attenuate mature pathological scarring.

On postoperative day 14, although HNA immunostaining revealed positive signals within the wound area, the staining pattern did not unequivocally indicate nuclear localization. Due to substantial non-specific background staining and incomplete co-localization with DAPI, these findings should be described as HNA immunoreactivity of uncertain specificity, rather than definitive evidence of transplanted human-cell persistence. The limited reliability of these signals raises the question of whether the therapeutic effects are mediated primarily through direct cell contribution or through paracrine signaling. While it is plausible that transplanted cells exert paracrine effects during the early phase of wound healing—secreting growth factors, cytokines, and extracellular vesicles that modulate the host microenvironment and recruit endogenous progenitor cells (Uminska et al., 2025; Bormann et al., 2023; Rajendran et al., 2026), this interpretation remains hypothetical, as the present study did not directly investigate paracrine signaling, evaluate secreted factors or extracellular vesicles, or perform lineage tracing. Future studies incorporating longitudinal cell tracking, lineage tracing, and analysis of paracrine mediators will be required to clarify the fate and mechanism of action of the transplanted cells. On postoperative day 21, PPAR-γ-positive sebaceous gland-like structures were detected in the hAAM + HFSCs + DPCs group, and epithelial bud-like structures morphologically suggestive of early follicular development were observed in some samples. PPAR-γ is a key regulator of sebaceous gland differentiation, and its positive expression supports the morphological identification of sebaceous gland-like structures (Wang et al., 2016). However, PPAR-γ is not a definitive marker of mature sebaceous glands; therefore, the PPAR-γ-positive structures observed in this study should be regarded as structures with morphological features resembling sebaceous glands rather than definitive sebaceous glands. Future studies should incorporate additional lineage-specific markers (e.g., CK15, LRIG1, GATA6, LGR6, trichohyalin) and functional assays to more rigorously characterize the identity, maturity, and functional relevance of these structures. Taken together, our findings suggest that the hAAM-based construct co-loaded with HFSCs and DPCs may support wound repair outcomes that more closely approximate native skin architecture, with preliminary morphological evidence of appendage-like formation. However, functional skin regeneration and definitive appendage regeneration were not demonstrated in the present study, and further investigation is required to validate these observations and establish the regenerative potential of this approach.

From a translational perspective, the use of autologous hair follicle-derived cells is a potential strategy for reducing immunological concerns associated with allogeneic cell transplantation. In principle, HFSCs and DPCs could be obtained from a small number of scalp hair follicles through minimally invasive procedures and expanded *ex vivo* before being incorporated into hAAM. However, the present study did not evaluate autologous cell isolation, expansion kinetics, therapeutic cell yield, or the time required to manufacture the cell-loaded construct. Therefore, the clinical feasibility and manufacturing timeline remain to be established. Future studies should optimize cell isolation and expansion protocols and determine whether sufficient numbers of HFSCs and DPCs can be generated within a clinically acceptable timeframe. Dermal fibroblasts and keratinocytes are indeed more readily accessible and can generally be expanded in larger quantities, making them attractive cell sources for conventional skin tissue engineering. However, the rationale for using HFSCs and DPCs in the present study was not simply to accelerate wound closure. Rather, we aimed to combine an epithelial progenitor population with a specialized hair follicle-associated mesenchymal population capable of providing inductive signals through epithelial–mesenchymal interactions (Toyoshima et al., 2012; Cao et al., 2025). DPCs have been implicated in hair follicle morphogenesis and HFSC activation, whereas HFSCs contribute to epithelial regeneration and appendage-associated differentiation (Lin et al., 2022; Correia et al., 2023). Therefore, the HFSC + DPC combination was selected to explore whether recreating aspects of the epithelial–mesenchymal niche could provide additional benefits beyond conventional keratinocyte/fibroblast-based wound repair. Nevertheless, our study did not directly compare HFSCs + DPCs with keratinocyte + fibroblast constructs, and therefore we cannot conclude that the former is superior. Direct comparative studies will be required to determine whether the use of hair follicle-derived cells provides a specific advantage in wound repair or appendage-associated regeneration.

This study has several limitations. First, we acknowledge that the gross wound closure observed, particularly in the hAAM + HFSCs + DPCs group, was largely driven by wound contraction, a well-recognized feature of murine excisional wound healing. Therefore, accelerated macroscopic closure should not be equated with true regenerative healing. Although the histological improvements and appendage-like structures suggest that the construct may positively modulate tissue quality beyond mere contraction, this limitation, together with the use of immunodeficient nude mice for xenogeneic transplantation, indicates that the observed healing responses may not fully reflect human wound repair. Second, although appendage-like structures were observed, their identity, functionality, and long-term fate remain unclear, and functional skin regeneration was not demonstrated. Moreover, due to limited tissue availability, additional lineage-specific markers (e.g., CK15, LRIG1, GATA6, trichohyalin, LGR6) recommended to confirm appendage identity could not be assessed in the present study. Therefore, we cannot definitively exclude the possibility that these structures represent non-specific epithelial aggregates. Third, the current study used HNA immunostaining to track transplanted cells, but the non-specific background and imperfect DAPI co-localization observed highlight the need for future studies utilizing high-resolution confocal microscopy, human-specific DNA-based detection methods, or lineage tracing to conclusively confirm the structural integration of the transplanted cells. Fourth, the sample size for the longitudinal wound closure analysis was limited to three animals per group. While this cohort size was sufficient to detect statistically significant differences in our repeated-measures ANOVA, we acknowledge that the small sample size limits the generalizability of the findings. Moreover, effect size estimates and *post hoc* power calculations derived from this small cohort may be unstable and should be interpreted with caution. Future studies with larger animal cohorts will be valuable to confirm these observations.

## 5 Conclusion

In summary, the hAAM-based combinatorial strategy for co-delivery of HFSCs and DPCs demonstrated favorable biocompatibility and was associated with improved wound closure, tissue repair, increased density of CD31-positive vascular structures, and altered extracellular matrix remodeling in a murine full-thickness skin wound model. In addition, appendage-like structures were observed in some samples, suggesting a potential role of this strategy in promoting skin tissue repair with preliminary evidence of appendage-like structures. However, the current study does not provide sufficient evidence to demonstrate functional skin regeneration, definitive appendage regeneration, or direct anti-fibrotic effects. Further studies incorporating lineage-specific characterization, long-term functional evaluation, mechanistic investigations, and the optimization of autologous cell sourcing and clinically feasible cell expansion will be necessary to determine the regenerative potential and translational value of this approach.

## Data Availability

The original contributions presented in the study are included in the article/Supplementary Material, further inquiries can be directed to the corresponding author.
