# Supplementary material for: Human acellular amniotic membrane loaded with hair follicle-derived cells promotes wound repair and appendage-like structure formation
Source: Front Cell Dev Biol. 2026 Oct 1;14:1947836. doi: 10.3389/fcell.2026.1947836 (PMC13669164; doi:10.3389/fcell.2026.1947836)
Supplement: Supplementary file 1 [file Supplementaryfile1.pdf]

Human Acellular Amniotic Membrane Loaded with Hair Follicle-Derived Cells Enhances Wound Repair and Appendage-Like Structure Formation

Supplementary Information

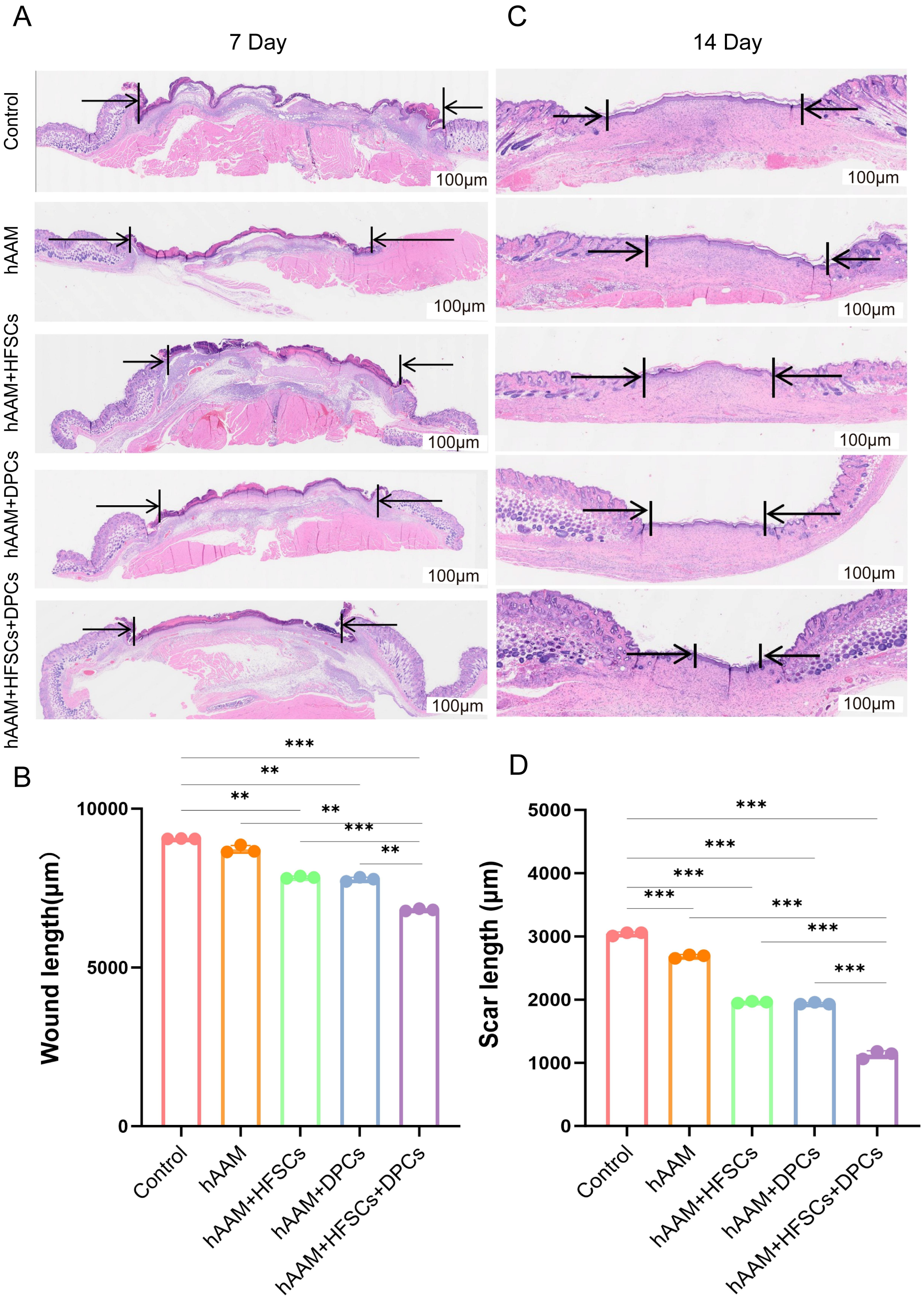

Supplementary Figure S1. Quantitative analysis of wound length and scar length during wound healing. (A) Representative H&E-stained whole-section images of wounds on postoperative day 7. Black arrows indicate the wound margins (the boundary between normal skin and the wound bed)

(scale bar: 100 μm). (B) Quantitative analysis of wound length on day 7, calculated as the distance between the two wound margins. (C) Representative H&E-stained whole-section images of wounds on postoperative day 14. Black arrows indicate the leading edges of the migrating neoepidermis and the distance between the two wound edges represents the residual scar length (scale bar: 100 μm). (D) Quantitative analysis of the scar length on day 14. Data are presented as mean ± SD (n = 3 animals per group). Statistical significance was assessed by one-way ANOVA followed by Tukey's post-hoc test. *$P < 0.05$, **$P < 0.01$, ***$P < 0.001$.

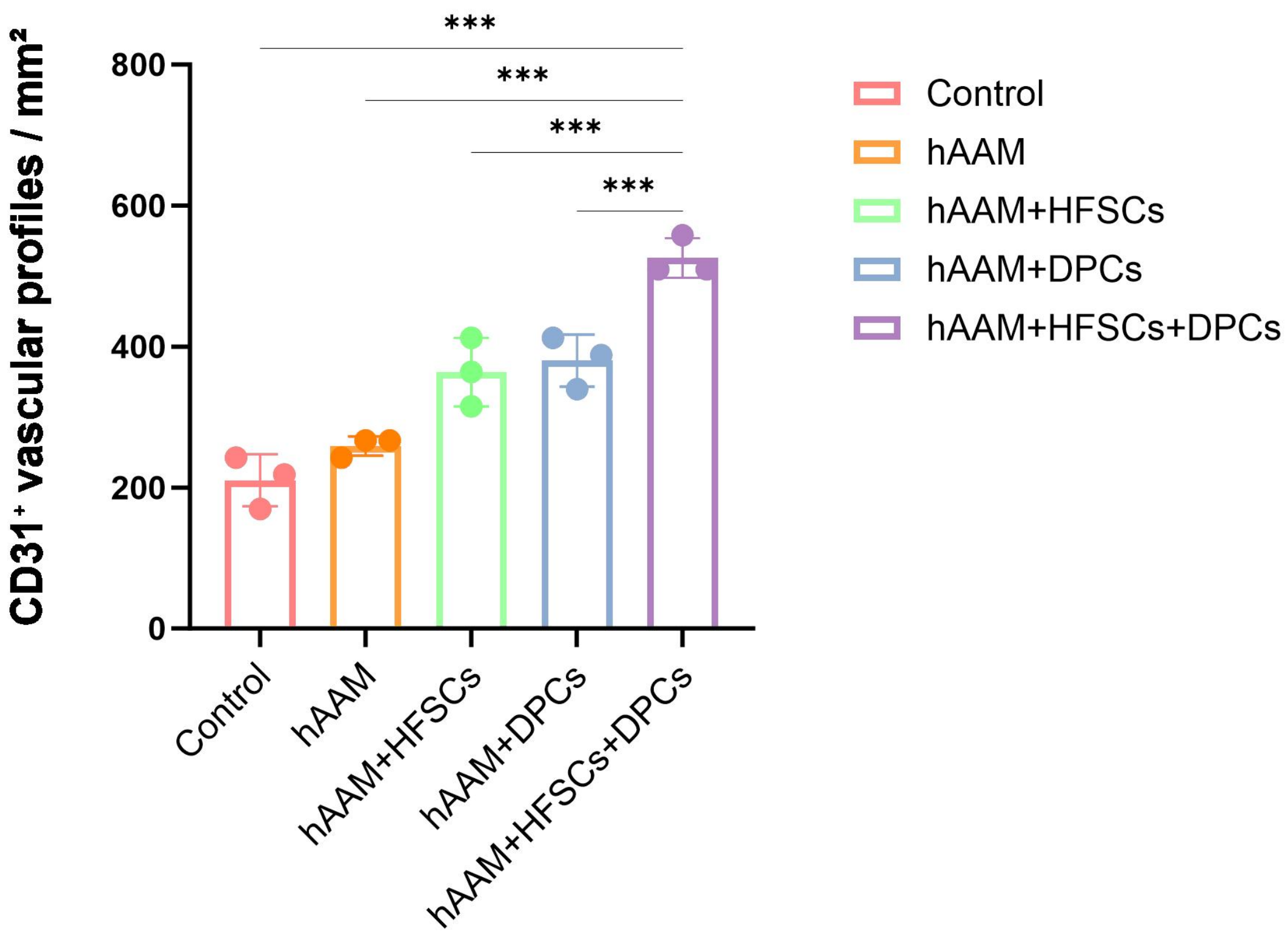

Supplementary Figure S2. Quantitative analysis of CD31-positive vascular structures in wound tissues on postoperative day 14. Vessel density was quantified as the number of CD31-positive vascular profiles per mm^2 using ImageJ software. Data are presented as mean ± SD (n=3 animals per group). Statistical significance was assessed by one-way ANOVA followed by Tukey's post-hoc test. ***$P < 0.001$

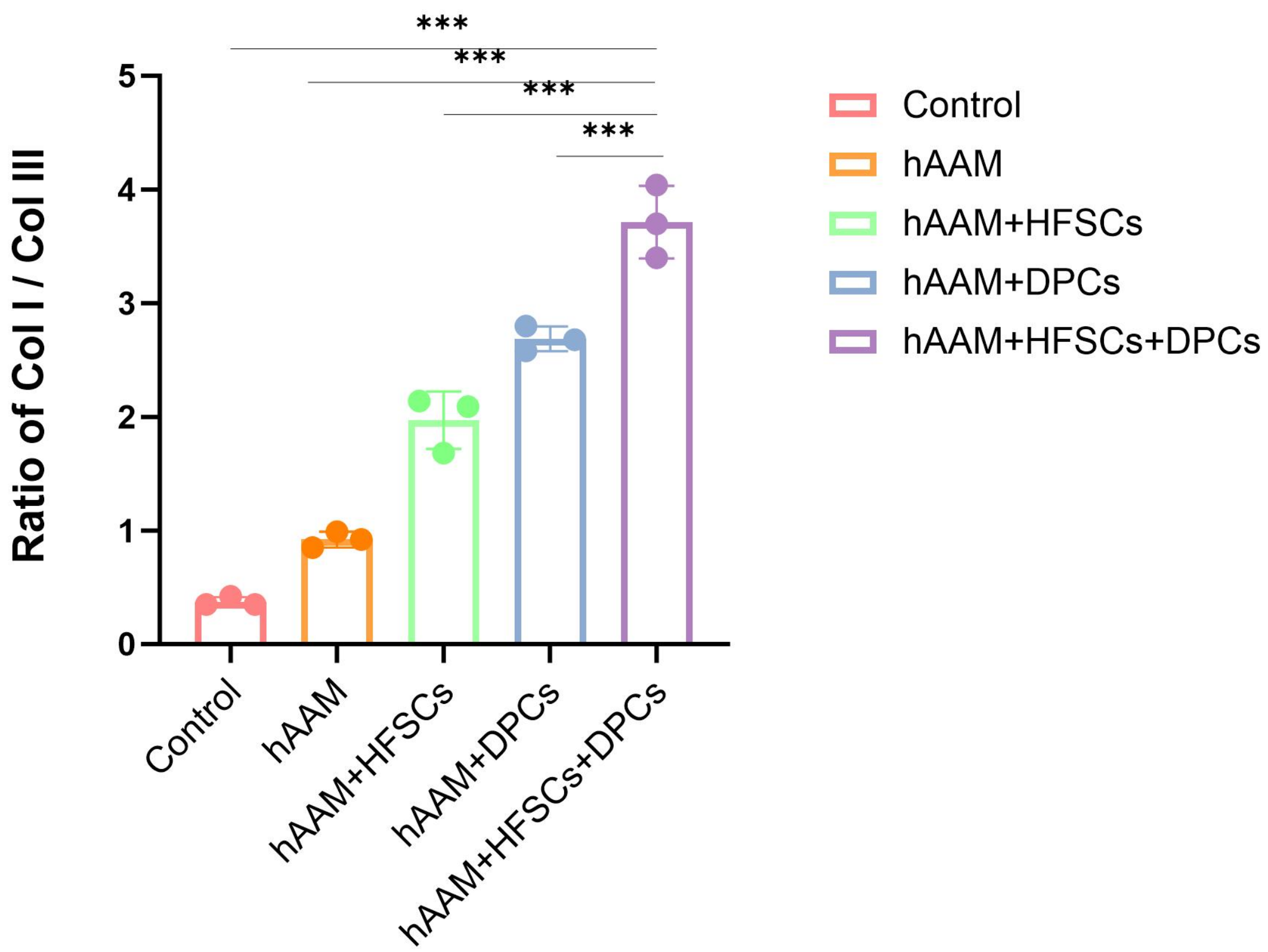

Supplementary Figure S3. Quantitative analysis of the Ratio of Col I/Col III . The Col I/Col III ratio was calculated for each animal on postoperative day 14. Data are presented as mean ± SD (n = 3 per group). ****P* < 0.001.

Supplementary Table S1. Results of repeated-measures ANOVA for wound closure rates.

Source	Type III Sum of Squares	df	Mean Square	F	P-value
Time	63195.237	2.031	31112.292	2795.971	< 0.001
Group	2148.022	4	537.005	114.159	< 0.001
Time × Group	416.859	8.125	51.307	4.611	0.002
Error (Time)	226.023	20.312	11.128		

Table S1 footnote: Greenhouse-Geisser correction was applied for within-subjects effects (Time and Time × Group) to adjust for violations of sphericity. df, degrees of freedom.

Supplementary Table S2. Quantitative assessment of appendage-like structures in Day 21 wound samples.

Group	Animal ID	PPARγ-positive sebaceous gland-like structures	epithelial bud like structures morphologically suggestive of early follicular structures
**hAAM+HFSCs+DPCs**	#1	3	2
	#2	4	7
	#3	2	1
	**Occurrence rate**	**3/3 (100%)**	**3/3(100%)**
**hAAM+DPCs**	#1	1	1
	#2	0	0
	#3	0	0
	**Occurrence rate**	**1/3 (33.3%)**	**1/3(33.3%)**

Note: n=3 per group. Data are presented as the absolute count of structures per whole wound section.
